# Is cerebral glucose metabolism related to blood–brain barrier dysfunction and intrathecal IgG synthesis in Alzheimer disease? A ^18^F-FDG PET/CT study: Erratum

**DOI:** 10.1097/01.md.0000503500.65089.86

**Published:** 2016-10-07

In the article “Is cerebral glucose metabolism related to blood–brain barrier dysfunction and intrathecal IgG synthesis in Alzheimer disease? A ^18^F-FDG PET/CT study”,^[1]^ which appeared in Volume 95, Issue 37 of *Medicine*, Dr. Francesco Ursini's name was incorrectly given. The article has since been corrected online.
